# Supplementary material for: Partially inserted nascent chain unzips the lateral gate of the Sec translocon
Source: EMBO Rep. 2019 Aug 5;20(10):e48191. doi: 10.15252/embr.201948191 (PMC6776908; doi:10.15252/embr.201948191)
Supplement: Supplementary file 2 — Expanded View Figures PDF [file EMBR-20-e48191-s002.pdf]

Expanded View Figures

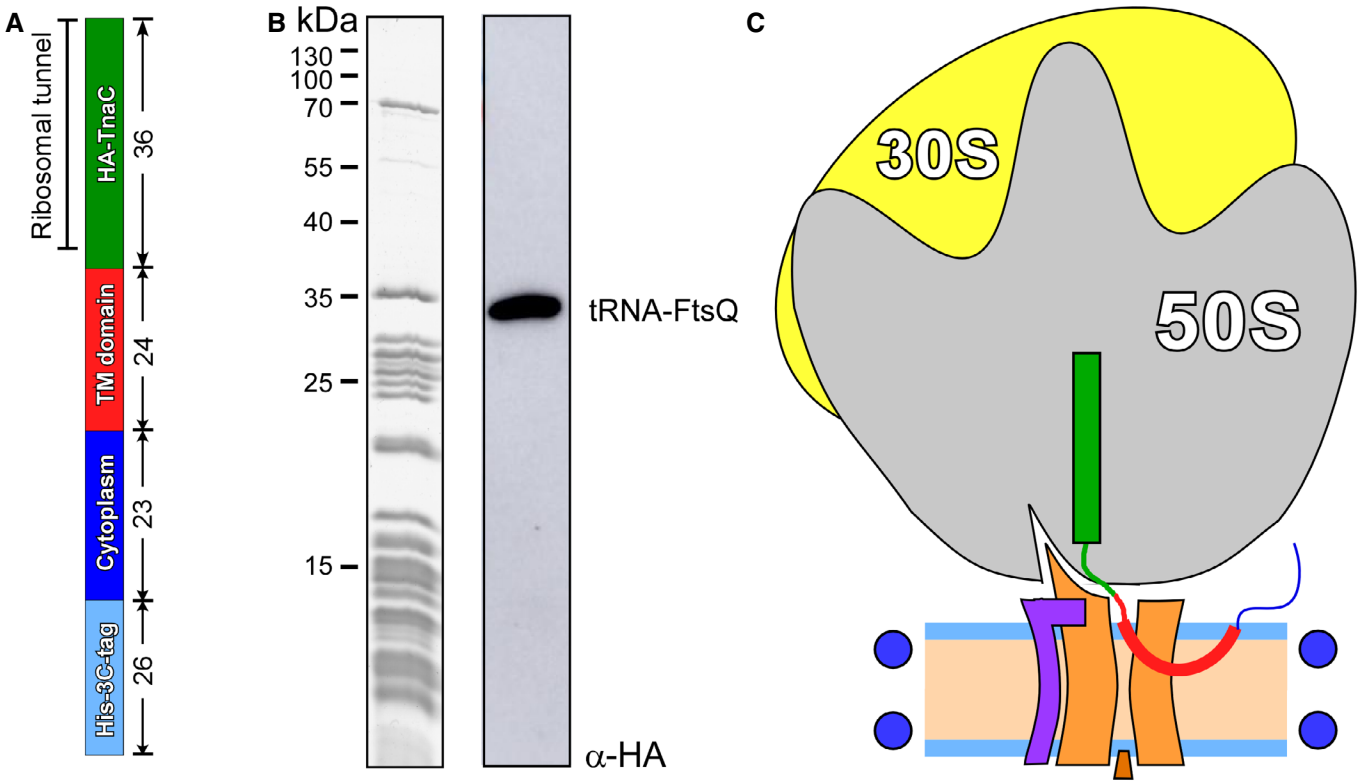

Figure EV1. Preparation of RNC FtsQ.

A The architecture of FtsQ nascent chain: N-terminal hexa-histidine tag was fused via 3C protease cleavage site to the N-terminal domain of FtsQ followed by hemagglutinin (HA) recognition tag and TnaC stalling sequence. The length (amino acids) of each segment is indicated.

B Coomassie-stained protein content of isolated RNC FtsQ (left) and Western blot against the HA tag within the nascent chain (right). Western blot visualizes a complex of the FtsQ nascent chain (109 aa, ~12 kDa) and tRNA (~25 kDa).

C Putative architecture of RNC:SecYEG-ND complex upon the insertion of the early intermediate of FtsQ.

© 2019 The Authors
