# Supplementary material for: Partially inserted nascent chain unzips the lateral gate of the Sec translocon
Source: EMBO Rep. 2019 Aug 5;20(10):e48191. doi: 10.15252/embr.201948191 (PMC6776908; doi:10.15252/embr.201948191)
Supplement: Supplementary file 1 — Appendix [file EMBR-20-e48191-s001.pdf]

Appendix

Partially inserted nascent chain unzips the lateral gate of the Sec translocon

Lukas Kater, Benedikt Frieg, Otto Berninghausen, Holger Gohlke, Roland Beckmann and Alexej Kedrov

Table of content | Page

Table of content	**1**
Appendix Figure S1. Resolution and angular distribution of RNC:SecYEG-ND cryo-EM data.	**2**
Appendix Figure S2. Comparison of translocon structures at dfferent stages of insertion.	**3**
Appendix Figure S3. Conformational dynamics of SecY subunit.	**4**
Appendix Figure S4. Conformational dynamics of the SecE subunit.	**5**
Appendix Figure S5. Interactions between SecY and SecE.	**6**
Appendix Figure S6. Cryo-EM reconstruction of SecYEG-ND:FtsQ RNC in presence of PE lipids.	**7**

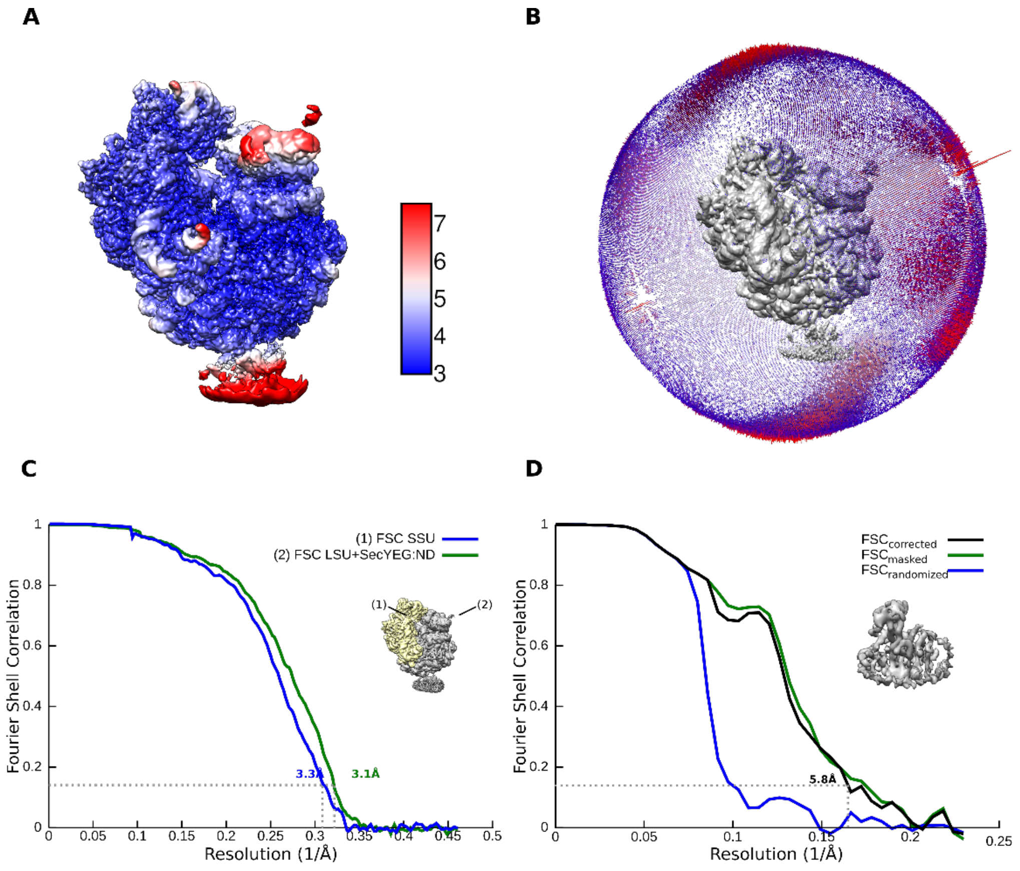

Appendix Figure S1. Resolution and angular distribution of RNC:SecYEG-ND cryo-EM data. (A) Local resolution map of the small subunit and large subunit including SecYEG-ND after the final unbinned multi-body refinement. (B) Relion angular distribution plot of the 70S:SecYEG-ND. (C) FSC curves for unbinned SSU and LSU:SecYEG-ND after multi-body refinement. (D) FSC curves for 2x binned SecYEG-ND after substraction of the ribosomal density, shifting SecYEG-ND to the center of mass and subsequent masked refinement.

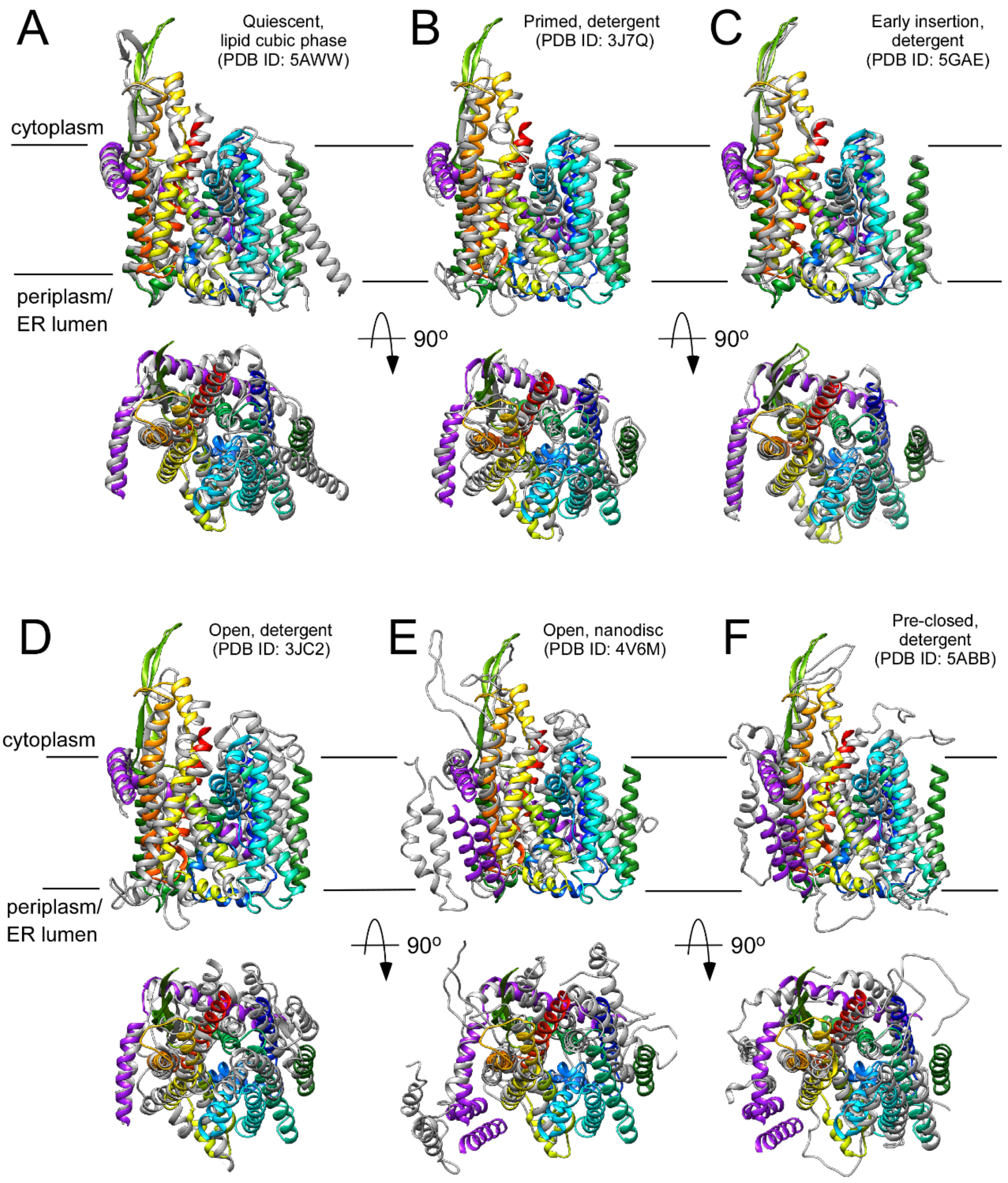

Appendix Figure S2. Comparison of translocon structures at dfferent stages of insertion. Superimposition of the resolved SecYEG-ND structure (colored) over previously known conformations of quiescent (A) and ribosome-bound translocons (B-F, shown in grey). The assigned state, molecular environment, and PDB access number are indicated above each superimposition.

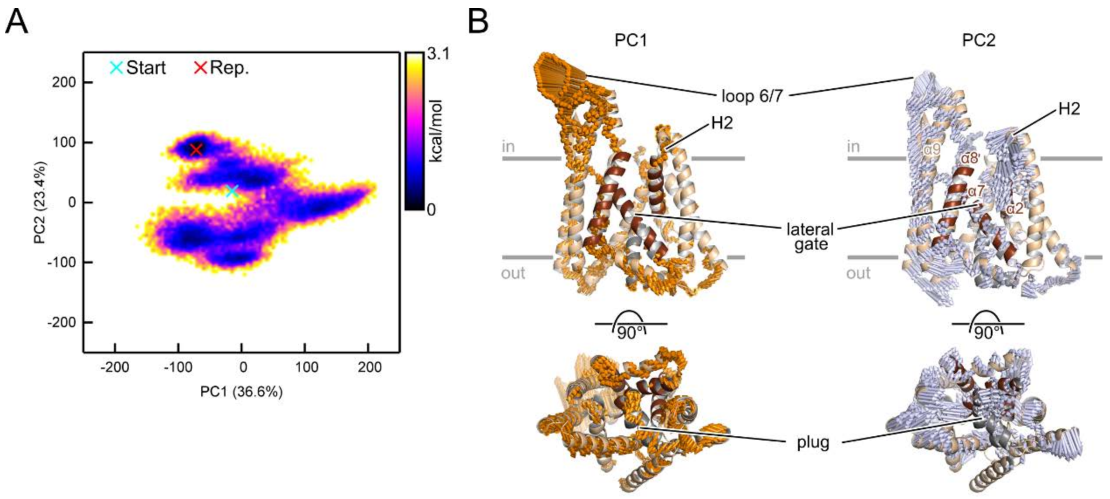

Appendix Figure S3. Conformational dynamics of SecY subunit. (A) Projection of conformations of joint, five, 1 µs long simulations of SecY onto the plane spanned by the first two principal components (PC); the conformations were color-coded according to their configurational free energy as computed by eq. 1. The numbers in parentheses on the X- and Y-axis denote the proportions of the total variance described by the respective PC. The MD starting structure is projected onto the free energy surface and labeled as cyan cross and the SecY structure closest to the energetic minimum as red cross. (B) Visualization of SecY displacements along the 1st (left; golden arrows – same as in Fig. 4C) and 2nd (right; silver arrows) PCs computed for the joint, five, 1 µs long simulations. Both PCs describe that motions of the loop 6/7 are correlated with motions of the cytoplasmic ends of SecY TMHs. The amplitudes of the motions were scaled, and a cutoff for small displacements was applied for best graphical representation. In the lower panels, the structures are rotated by 90° such that the periplasmic plug is now oriented towards the viewer.

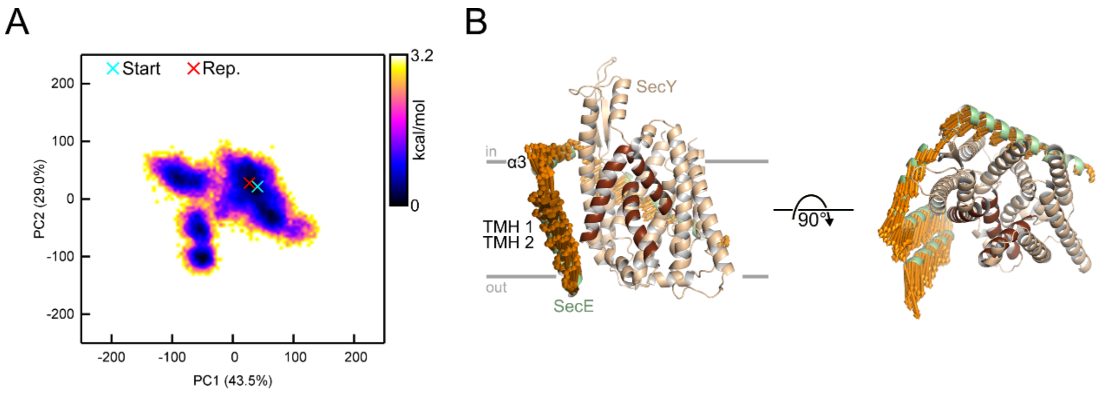

Appendix Figure S4. Conformational dynamics of the SecE subunit. (A) Projection of conformations of joint, five, 1 µs long simulations of SecE onto the plane spanned by the first two principal components (PCs); the conformations were color-coded according to their configurational free energy as determined by eq. 1. The numbers in parentheses on the X- and Y-axis denote the proportion of the total variance described by the respective PC. The MD starting structure is projected onto the free energy surface and labeled as cyan cross and the SecE structure closest to the energetic minimum as red cross. (B) Visualization of displacements along the 1st (golden arrows) PC computed for the joint, five, 1 µs long simulations. The amplitudes of the motions were scaled, and a cutoff for small displacements was applied for best graphical representation. In the right panel, the structure is rotated by 90° such that the periplasmic plug is now oriented towards the viewer. The left panel (side view) is the same, as in Fig. 4F.

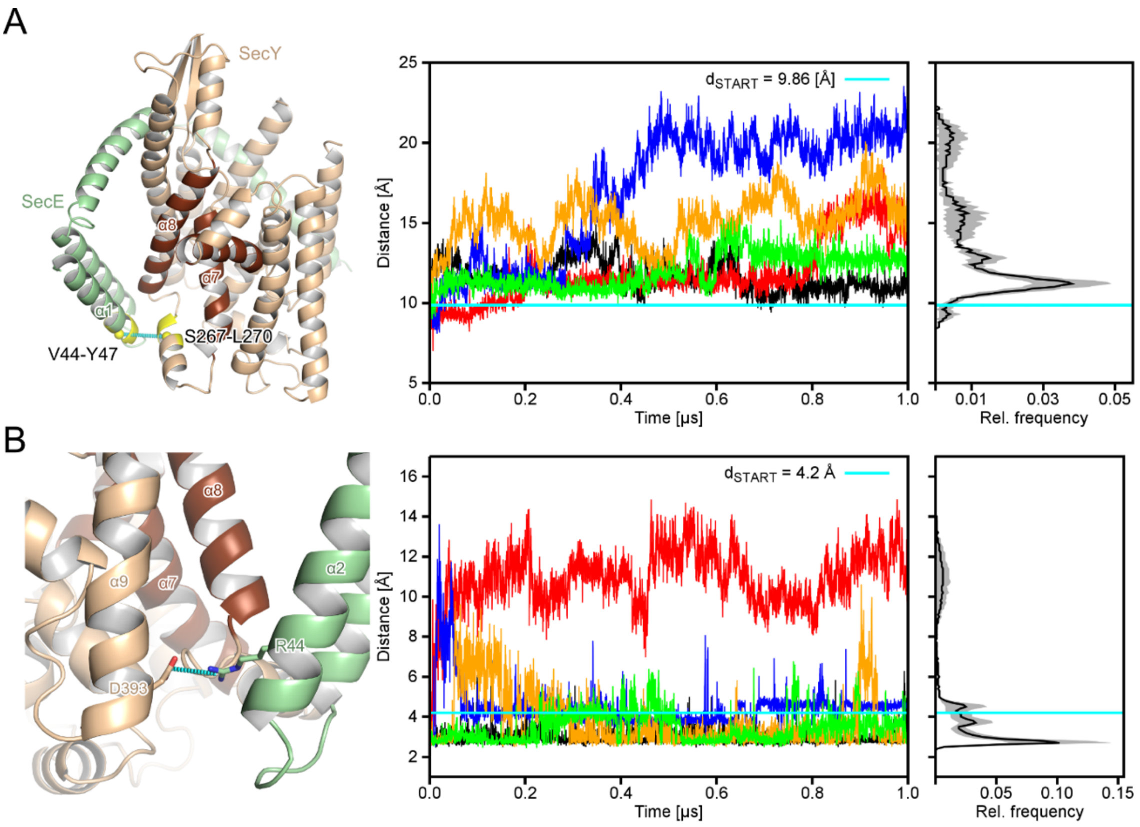

Appendix Figure S5. Interactions between SecY and SecE. (A) Distance of the closest contact to characterize the interaction between SecY and SecE at the periplasmic interface. The distance was measured between the backbone centers of mass of residues S267 – L270 on a periplasmic helix connecting TMHs 7 ("α7") and 8 ("α8") in SecY and V44 – Y47 of TMH 1 ("α1") in SecE. (B) Complementary, a distance was measured between the centers of mass of the functional groups of D393 (SecY) and R44 (SecE). In (A) and (B), the middle panels show the distances as a function of the simulation time for five MD simulations (differently colored lines) and the right panels the mean relative frequencies (n = 5) plotted as a histogram. The gray areas denote the standard error of the mean for each bin. The cyan lines denote the distances in the starting structure

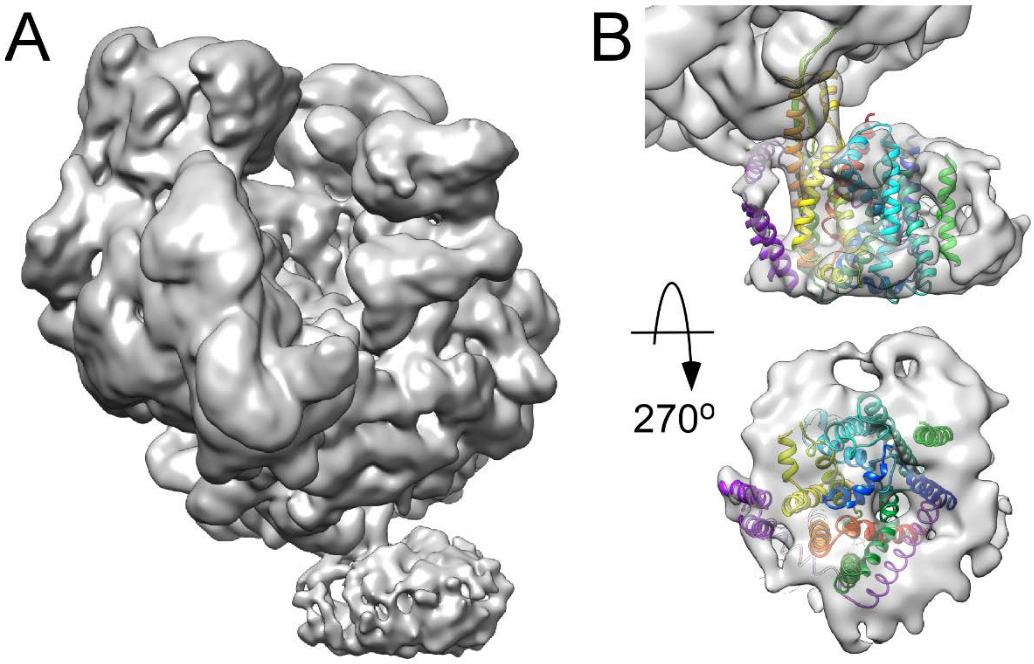

Appendix Figure S6. Cryo-EM reconstruction of SecYEG-ND:FtsQ RNC in presence of PE lipids. (A) Cryo-EM density map of the RNC-bound translocon in nanodiscs composed of 30 mol % POPG, 30 mol % POPE, and 40 mol % POPC. The map is filtered to 9 Å. (B) An overlay of the cryo-EM density of SecYEG-ND in (A) and the molecular model of SecYEG built for POPG/POPC-based nanodiscs. Despite the overall agreement of the structures, substantially lower resolution could be achieved in presence of POPE lipids, suggesting a higher sample heterogeneity and flexibility.
